# Assessment of a portable lactate meter for field use in the white rhinoceros (*Ceratotherium simum*)

**DOI:** 10.4102/ojvr.v84i1.1399

**Published:** 2017-11-10

**Authors:** Georgina C. Cole, Adrian S.W. Tordiffe, Gerhard Steenkamp

**Affiliations:** 1 Department of Companion Animal Clinical Studies, University of Pretoria, South Africa; 2 Department of Paraclinical Sciences, University of Pretoria, South Africa

## Abstract

Blood lactate is a predictor of mortality in critically ill humans and animals. Handheld lactate meters have the potential to be used in the field to evaluate the condition of severely injured rhinoceroses but have not been compared with laboratory-based methods. Agreement between a handheld lactate meter and a laboratory method was assessed, as was the stability of rhino blood lactate in the anticoagulant sodium fluoride/potassium oxalate (fluoride/oxalate). Blood samples were obtained from 53 white rhinos that had been immobilised for management reasons. Lactate was measured by means of a handheld meter using whole blood in heparin (WBHEP), whole blood in fluoride/oxalate (WBFO) and fluoride/oxalate plasma (PFO). Results were recorded in both blood (BL) and plasma (PL) modes and compared to an established laboratory method for measuring plasma lactate. To assess the stability of lactate over time, blood lactate in fluoride/oxalate was measured on the handheld meter at intervals for up to 91 h. Agreement was best using WBFO in PL mode, with small bias (−0.16), tight 95% limits of agreement (LOA) (−1.46, 1.14) and a Pc (95% CI) of 0.97 (0.92, 0.99). The agreement was improved for all sample types when using the PL mode compared to the blood lactate (BL) mode. Blood lactate was stable in fluoride/oxalate for 91 h, with a mean change from baseline of 0.15 (−0.178, 0.478) mmol/L (mean, 95% CI). The handheld meter was found to be suitable for field use in white rhinos but provided more reliable results with the device in PL mode. Furthermore, rhino blood lactate was found to be stable in fluoride/oxalate for as long as 3 days.

## Introduction

Lactate is produced during anaerobic glycolysis where the conversion of pyruvate to lactate facilitates continued adenosine triphosphate (ATP) production in the absence of oxygen (Lagutchik et al. 1996). Increased blood lactate levels may occur as a result of decreased oxygen delivery in circulatory or septic shock, increased oxygen demands such as exercise or shivering, or decreased oxygen utilisation in conditions such as systemic inflammatory response syndrome (SIRS) or renal failure (Cohen, Woods & Krebs 1976; Fall & Szerlip 2005; Huckabee 1961a, 1961b).

Blood lactate measurement in human patients has been extensively studied and demonstrated to be an effective predictor of mortality and useful in monitoring response to therapy in numerous critically ill patient groups (Aslar et al. 2004; Cerovic et al. 2003; Fall & Szerlip 2005; Lee D Cady et al. 1973; McNelis et al. 2001; Nguyen et al. 2004). Lactate measurement in horses is predictive of mortality in sick neonates (Borchers et al. 2012; Corley, Donaldson & Furr 2005; Henderson et al. 2008) and adult emergencies (Delesalle et al. 2007; Furr, Lessard & White 1995; Johnston, Holcombe & Hauptman 2007; Schulman, Nurton & Guthrie 2012; Tennent-Brown et al. 2010). In bovine medicine, lactate can be useful as a prognostic indicator in cattle with respiratory disease (Coghe et al. 2000; Helena et al. 2015) and abomasal disorders (Buczinski, Boulay & Francoz 2015; Figueiredo et al. 2008). In dogs, lactate measurement has prognostic value in systemically ill animals (Lagutchik et al. 1998; Stevenson et al. 2007b), dogs with hypotension (Ateca, Dombrowski & Silverstein 2015), septic peritonitis (Cortellini, Seth & Kellett-Gregory 2014) and babesiosis (Nel et al. 2004). Lactate is also a predictor of gastric necrosis and outcome in dogs with gastric dilatation-volvulus (De Papp, Drobatz & Hughes 1999).

Rhino poaching has increased dramatically in South Africa in recent years, with the number of rhinos killed rising from 13 in 2007 to 1215 in 2014 (Department of Environmental Affairs 2015). Rhinos that survive a poaching event may have serious injuries such as gunshot wounds to the head, abdomen, thorax and limbs, or systemic complications because of prolonged immobilisation with synthetic opioids. These animals may also have severe facial injuries with open sinuses and exposed bone resulting from traumatic horn removal. Other injuries such as fractures of the axial or appendicular skeleton and muscle trauma have been reported.

Little is known about the assessment of critically injured rhinos or how to treat them. The practical difficulties associated with treating wild animals for severe injuries make major interventions, intensive care and long-term analgesia challenging. Identifying prognostic indicators in critically injured rhinos that have initially survived a poaching event may help to focus treatments in injured animals as well as monitor response to therapy. It may improve the welfare of injured rhinos in those cases where long-term survival is unlikely, and euthanasia may be more appropriate. It could also potentially enable more effective use of resources so that time and money can be directed towards cases with a higher probability of success and may help identify areas for future research.

The use of blood lactate as a prognostic indicator in rhino species has not been investigated. Laboratory measurement of blood lactate requires special sample handling. Blood samples must be kept at 4 °C and plasma separated almost immediately to prevent falsely elevated lactate levels occurring from continued cellular glycolysis (Sacks 2008). Rhinos in South Africa are mostly free ranging and are kept in game reserves or on private property that is often remote, making this type of sample handling impractical. Handheld lactate meters designed for human use have been validated in a number of animal species making animal-side lactate measurement feasible and practical in emergency settings (Buczinski et al. 2014; Burgdorf-Moisuk et al. 2012; Delesalle et al. 2007; Evans & Golland 1996; Stevenson et al. 2007a; Tennent-Brown et al. 2007; Thorneloe, Bédard & Boysen 2007; Tynan et al. 2015). Results are available in minutes allowing treatment decisions based on results to be made at the animal’s side. This is especially pertinent in a free-ranging species where a diagnosis must be made and treatments administered within the short period that the animal is immobilised.

The Roche Accutrend plus system (Roche Diagnostics Ltd. CH-6343 Rotkreuz, Switzerland) is a portable handheld device that measures blood lactate and has been designed for use in human healthcare. It has been evaluated and found to be accurate for use in horses (Tennent-Brown et al. 2007), dogs (Acierno & Mitchell 2007; Karagiannis et al. 2013) and cats (Acierno et al. 2008). Its use in the rhino has not been evaluated. Accordingly, the aim of this study was to evaluate a handheld lactate meter for field use in the white rhino by comparing it with a laboratory bench-top analyser. The effects of sample type (blood versus plasma) and anticoagulant (heparin versus sodium fluoride/potassium oxalate) on the agreement of the handheld device with the laboratory method were also investigated.

The Accutrend measures L-lactate in a drop of blood applied to a disposable test strip. The test strips are made up of several layers. The top layer is a mesh, and below that is glass fibre where leucocytes and erythrocytes are filtered out. Plasma enters the bottom layer of the strip where lactate oxidase converts lactate to pyruvate using the electron carrier bis-[2-hydroxyethyl]-4-hydroxyiminocyclohexa-2, 5-dienylidene ammonium chloride (Pennell & Tracy 1999):

$$\text{Lactate}+\text{electron carrier}\overset{\text{Lactate oxidase}}{\to }\text{pyruvate}+\text{electron carrier}(\text{reduced})$$ [Eqn 1]

A further chemical reaction then occurs where the reduced electron carrier reacts with phosphomolybdic acid to form the dye molybdenum blue. The colour intensity of the dye is measured by reflectance photometry, and the L-lactate concentration is calculated (Stevenson et al. 2007a; Tennent-Brown et al. 2007).

A result appears in the digital display in 60 s. The Accutrend can be set to read either blood lactate (BL mode) or plasma lactate (PL mode). The machine measures plasma lactate concentration, and an algorithm is used to calculate whole blood lactate from this value. The algorithm is specific for human blood and may not give accurate results when used in other species.

The Cobas Integra 400 Plus bench-top analyser (Roche, F. Hoffmann-La Roche Ltd Grenzacherstrasse 1244070 Basle, Switzerland) is a laboratory-based analyser that measures lactate in 192 µL of plasma that is automatically pipetted from a sample by the machine. The manufacturer’s instructions state that either heparinised plasma or plasma in sodium fluoride/potassium oxalate (fluoride/oxalate) may be used. The test principle is an enzymatic colorimetric method. It is an established method of laboratory-based lactate detection in humans (De Backer 2003; Toffaletti et al. 1992) and is currently used to measure lactate in a variety of mammalian species at the Onderstepoort Veterinary Academic Hospital.

Lactate levels will increase in blood samples after collection unless plasma is immediately separated or antiglycolytic agents are used. A further aim of this study was therefore to assess the stability of rhino blood lactate in the antiglycolytic agent fluoride/oxalate over time.

## Materials and methods

### Study population and capture

Study subjects were 53 white rhinos (*Ceratotherium simum*) from three locations in South Africa: 48 animals were from a game farm with a large breeding population of white rhinos (reserve 1) and three from a private game reserve with a small population (less than 10) of rhinos (reserve 2). Furthermore, two white rhinos from another private game reserve (reserve 3) were included in the lactate stability study only. Animals were immobilised for management reasons such as translocation, horn removal or both. On reserves 1 and 3, rhinos were darted from a vehicle. Supplementary feeding from vehicles was routinely carried out, meaning that the animals were habituated and that darting was carried out with minimal pre-capture stress or activity. The three rhinos in reserve 2 were darted from a helicopter. Rhinos were darted with a combination of etorphine (Captivon 9.8 mg/mL, Wildlife Pharmaceuticals South Africa, Karino) and azaperone (Stresnil, 40 mg/mL, Schering Plough Animal Health, Kenilworth, New Jersey, USA). Butorphanol (20 mg/mL, Kyron Laboratories, Johannesburg, South Africa) was given intravenously once rhinos were recumbent to improve blood oxygenation. Drug dosages used were standard for rhino capture (Burroughs et al. 2012) and calculated according to the size of the animal which was estimated by the veterinarian in charge of immobilisation. Rhinos were put into life-stage categories based on age (if known) or size and breeding activity (if actual age unknown): Calf (< 1 year), yearling (1–2 years), sub-adult (2–7 years) and adult (> 7 years). Ambient temperatures ranged from 22 °C to > 35 °C, and immobilisation took place during daylight hours.

### Blood sampling and lactate measurement

Blood samples were taken from the auricular vein, or occasionally the radial vein in the case of calves. Blood was collected in evacuated tubes (Vacuette^®^ and Vaccutainer^®^, Beckton, Dickinson & Co, New Jersey, USA) containing lithium heparin and also tubes containing fluoride/oxalate using an 18G double-ended vacuum tube needle. Lactate was measured on the Accutrend within 5 min of blood collection using either heparinised whole blood (WBHEP) or whole blood in fluoride/oxalate (WBFO) by placing one drop of fresh blood on to the lactate test strip according to the manufacturer’s instructions. To ascertain which type of sample (blood or plasma) and which mode on the Accutrend (BL or PL) would give results in closest agreement to those of the bench-top analyser, both whole blood (in the field) and plasma (in the laboratory) were used, and results were recorded in both BL and PL modes. This is similar to the method used by Tennent-Brown et al, assessing the accuracy of Accutrend in horses (Tennent-Brown et al. 2007). The manufacturer’s instructions state that only fresh blood or heparinised whole blood should be used on the Accutrend. In this study, WBHEP, WBFO and fluoride/oxalate plasma (PFO) were used. Regular control checks were performed throughout the study using lactate control solutions according to the manufacturer’s instructions to ensure proper functioning of the instrument. A glass Pasteur pipette with a rubber dropper was used to apply blood or plasma to the test strip throughout the study. Blood tubes were placed in an upright position in a cooler bag containing ice blocks and left to stand.

To test the stability of rhino blood lactate in fluoride/oxalate, lactate from eight blood samples collected into fluoride/oxalate tubes as described above was measured on the Accutrend at various time intervals. Blood tubes were not inverted each time, allowing separation of the plasma and cellular component of blood to occur with gravity, and plasma was aspirated from the top of the tube for each lactate measurement. Therefore, whole blood was used for the first measurement (*t* = 0) and plasma for all subsequent measurements.

For the remaining samples, plasma from the fluoride/oxalate tubes was separated from the cellular fraction by centrifugation within 8 h of collection and frozen at −20 °C. Samples were then transferred on ice to the Onderstepoort Veterinary Academic Hospital and stored at −80 °C (within 48 h of collection) for analysis at a later date. At the time of analysis, PFO from all study subjects was removed from the freezer and defrosted at room temperature. Plasma lactate for each rhino was then measured concurrently with the Accutrend and with the Cobas bench-top analyser. Daily quality controls and calibrations were run as part of the laboratory’s standard protocols.

### Statistics

Comparisons made between lactate measured on the Accutrend and the Cobas bench-top analyser were analysed using Bland Altman statistical analysis (Bland & Altman 1986), Passing and Bablok regression analysis (Passing & Bablok 1983) and concordance correlation (Lawrence & Lin 1989). All calculations were performed using MedCalc^®^ Version 12.2.0.0.

## Results

For comparison of lactate measurement techniques, blood samples were taken from 51 rhinos (48 from reserve 1 and 3 from reserve 2) consisting of 37 females and 14 males. Sampling of some individuals on consecutive days allowed for greater than 51 comparisons between groups (*n* = 57). Of the female rhinos 65% were adults, 11% sub-adult, 13% juvenile and 11% calves. Of the male rhinos, 50% were adult, 14% sub-adult, 7% juvenile and 29% calves. The Cobas bench-top analyser has a measuring range of 0.2 mmol/L to 15.5 mmol/L. Lactate measured on the Cobas bench analyser (*n* = 57) ranged from 0.9 to 14.3, mean 4.6 (mmol/L). The Accutrend reads lactate values from 0.8 mmol/L to 21.7 mmol/L in BL mode and 0.7 mmol/L to 26 mmol/L in PL mode. Lactate measured with the Accutrend for all sample types combined ranged from 0.9 mmol/L to 13.8 mmol/L in BL mode and 0.8 mmol/L to 16.5 mmol/L in PL mode.

Plasma lactate measured with the Cobas bench-top analyser (COBAS) was compared with lactate measured with the Accutrend for each of the following sample types and meter settings: Heparinised whole blood with the Accutrend in BL mode (WBHEP BL) and in PL mode (WBHEP PL), fluoride/oxalate whole blood with the Accutrend in BL and PL mode (WBFO BL, WBFO PL), and plasma in fluoride/oxalate with the Accutrend in BL and PL mode (PFO PL, PFO BL).

**FIGURE 1:**
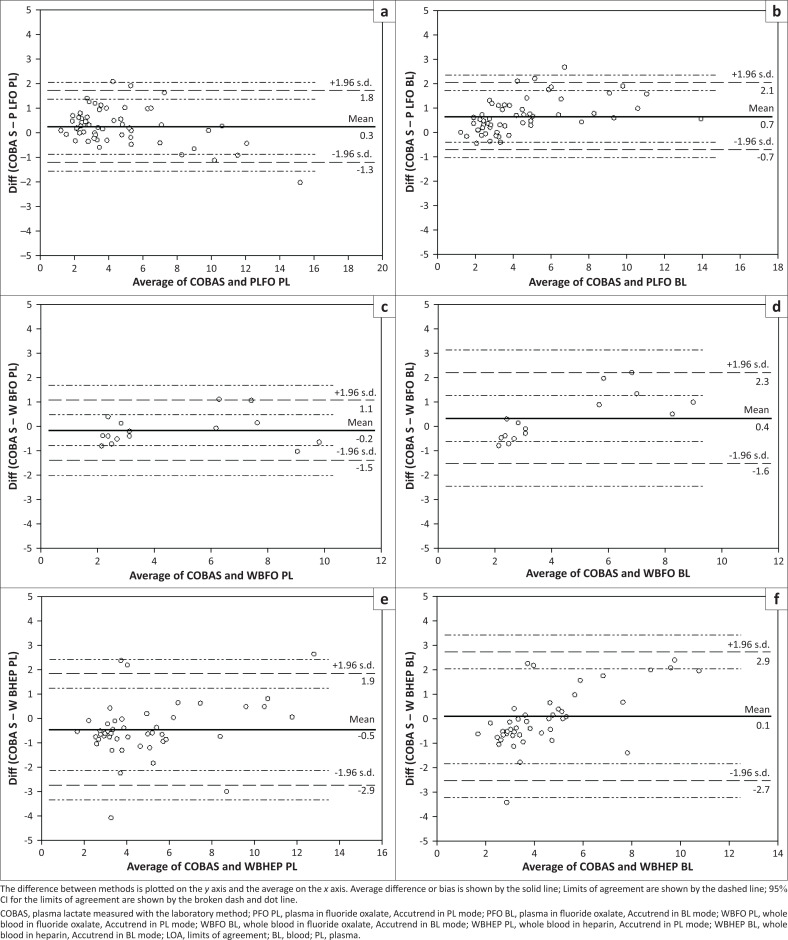
Bland – Altman plots of rhino blood lactate concentration. The plots describe agreement between plasma lactate measured with a laboratory method (COBAS) and the Accutrend handheld meter. Lactate is measured on the Accutrend with different sample types using either the blood or plasma mode. (a) Plasma in fluoride/oxalate, plasma mode, (b) plasma in fluoride/oxalate, blood mode, (c) whole blood in fluoride/oxalate, plasma mode, (d) whole blood in fluoride/oxalate, blood mode, (e) whole blood in heparin, plasma mode and (f) whole blood in heparin, blood mode.

Bland–Altman analysis (Bland & Altman 1986) assesses agreement between two methods by plotting the differences between paired measurements against the average of those measurements. The magnitude of the bias (average of differences) describes the level of agreement, (0 = perfect agreement). The limits of agreement (95% LOA) are bias plus or minus 1.96 x standard deviation (+ - 1.96 s.d.) and describe where 95% of the differences between the measurement techniques are likely to lie. LOA refer to actual measurements of the technique in question (lactate in mmol/L) and if they encompass differences that are not clinically significant then the two methods may be considered to agree. Bland–Altman analysis indicated good agreement between all group comparisons with a mean difference close to zero for all comparisons over the whole range of lactate values (Figure 1). When the Accutrend was compared to the Cobas bench-top analyser the smallest mean differences were seen when WBHEP was used in the BL mode and when WBFO was used in the PL mode (0.11 and −0.16 respectively). The 95% LOA were narrowest when WBFO was used in the PL mode, but widest when WBHEP was used in the BL mode.

Concordance analysis results are displayed in Table 1 as concordance correlation coefficient *Pc* and 95% CI. Highest level of agreement (*Pc*, 95% CI) was seen between the comparisons COBAS – WBFO PL and COBAS – PFO PL with values of 0.97 (0.92, 0.99) and 0.96 (0.94, 0.98) respectively. Poorest agreement, with *Pc*, 95% CI values of 0.89 (0.82, 0.94) and 0.85 (0.78, 0.90) was seen when lactate measured on the Cobas bench-top analyser was compared with lactate measured on the Accutrend in PL and BL mode using WBHEP.

**TABLE 1:** Bland – Altman, Passing and Bablock and Concordance analysis of plasma lactate measured with a laboratory method compared with the Accutrend handheld meter. Lactate is measured on the Accutrend with different sample types using either the blood or plasma mode.

Comparison	COBAS – PFO PL	COBAS – PFO BL	COBAS – WBFO PL	COBAS – WBFO BL	COBAS – WBHEP PL	COBAS – WBHEP BL
Observations (*n*)	57	57	15	15	46	46
Bland–Altman analysis						
Average difference mmol/L (95%LOA)	0.28	0.70	−0.16	0.35	−0.47	0.11
	(−1.25, 1.82)	(−0.75, 2.15)	(−1.46, 1.14)	(−1.62, 2.32)	(−2.89, 1.94)	(−2.66, 2.89)
Passing and Bablok regression						
Intercept A 95% CI	(0.18, 0.69)	(−0.44, 0.17)	(−1.52, −0.01)	(−2.94, −0.71)	(−1.54, −0.58)	(−2.86, −1.53)
Slope B 95% CI	(0.86, 1.02)	(1.10, 1.30)	(0.94, 1.35)	(1.20, 1.87)	(0.98, 1.23)	(1.35, 1.69)
Cusum test for linearity	*p* < 0.01	*p* < 0.01	*p* < 0.01	*p* < 0.01	*p* < 0.01	*p* < 0.01
Concordance correlation coefficient	0.96	0.94	0.97	0.92	0.89	0.85
*Pc* (95% CI)	(0.94, 0.98)	(0.90, 0.96)	(0.92, 0.99)	(0.83, 0.96)	(0.82, 0.94)	(0.78, 0.90)

COBAS, plasma lactate measured with the laboratory method; PFO PL, plasma in fluoride oxalate, Accutrend in PL mode; PFO BL, plasma in fluoride oxalate, Accutrend in BL mode; WBFO PL, whole blood in fluoride oxalate, Accutrend in PL mode; WBFO BL, whole blood in fluoride oxalate, Accutrend in BL mode; WBHEP PL, whole blood in heparin, Accutrend in PL mode; WBHEP BL, whole blood in heparin, Accutrend in BL mode.

When Passing and Bablok regression was performed, the 95% confidence intervals for the intercept A did not include the value zero for all, apart from the comparison COBAS – PFO BL indicating that systematic differences occur between measurements for all other comparisons (Table 1). For slope B the 95% CI did not include the value one for the following comparisons: COBAS – PFO BL, COBAS – WBFO BL and COBAS – WBHEP BL; indicating that proportional differences exist between the measurements. There were no comparisons where both A and B were not significantly different from zero and one, and the Cusum linearity test yielded *p* < 0.01 for all comparisons indicating deviation from linearity.

Comparisons between methods were split according to lactate concentration to see if agreement was improved at lower lactate concentrations. This was not done for comparisons COBAS – WBFO BL and COBAS – WBFO PL because of the small sample size. Results are shown in Table 2. Bland–Altman agreement was improved with both a smaller mean difference and narrower 95% LOA when lactate was < 5 mmol/L for the comparison COBAS – PFO BL. Smaller 95% LOA but slightly larger mean differences were seen for comparisons COBAS – WBHEP BL, COBAS – WBHEP PL and COBAS – PFO PL when lactate concentration was < 5 mmol/L. Values of *Pc* were lower, and 95% CI wider, for all groups when concordance analysis was performed on comparisons split according to lactate concentration < 5 mmol/L and ≥ 5 mmol/L. Improved agreement was seen with Passing and Bablok regression when lactate < 5 mmol/L for the following comparisons: COBAS – PFO BL, COBAS – PFO PL and COBAS – WBHEP PL, where intercept A and slope B were not significantly different from zero and one, indicating no systematic or proportional differences, whereas for lactate ≥ 5 mmol/L the 95% CI for intercept A and slope B did not include zero and one. However, *p* was still less than 0.01 for all groups, indicating poor fit of a linear model.

**TABLE 2:** Bland – Altman, Passing and Bablock and Concordance analysis of plasma lactate measured with a laboratory method compared with the Accutrend handheld meter with analysis split according to laboratory method lactate concentration < or ≥ 5 mmol/L. Lactate is measured on the Accutrend with different sample types using either the blood or plasma mode.

Comparison	COBAS – PFO PL		COBAS – PFO BL		COBAS – WBHEP PL		COBAS – WBHEP BL	
	< 5 mmol/L	≥ 5 mmol/L	< 5 mmol/L	≥ 5 mmol/L	< 5 mmol/L	≥ 5 mmol/L	< 5 mmol/L	≥ 5 mmol/L
Observations (*n*)	38	19	38	19	31	15	31	15
Bland Altman analysis								
Average difference mmol/L (95%LOA)	0.34 (−0.70, 1.38)	0.2 (−2.1, 2.4)	0.37 (−0.60, 1.34)	1.36 (−0.05, 2.76)	−0.7 (−2.8, 1.3)	0.1 (−2.7, −2.9)	−0.5 (−2.3, 1.3)	1.4 (−1.50, 4.2)
Passing and Bablok regression								
Intercept A 95%CI	(−0.07, 0.88)	(0.97, 2.94)	(−0.70, 0.48)	(0.55, 2.65)	(−1.41, 0.25)	(−4.13, 1.42)	(−3.06, −0.64)	(−4.07, 1.42)
Slope B 95% CI	(0.77, 1.16)	(0.66, 0.89)	(0.94, 1.44)	(0.83, 1.11)	(0.71, 1.22)	(0.88, 1.52)	(1.03, 1.8)	(1.05, 1.8)
Cusum test for linearity	*p* < 0.01	*p* < 0.01	*p* < 0.01	*p* < 0.01	*p* < 0.01	*p* < 0.01	*p* < 0.01	*p* < 0.01
Concordance analysis								
*Pc* (95% CI)	0.81 (0.69, 0.90)	0.93 (0.87, 0.96)	0.79 (0.66, 0.88)	0.85 (0.73, 0.93)	0.42 (0.15, 0.63)	0.88 (0.68, 0.95)	0.45 (0.17, 0.66)	0.74 (0.49, 0.87)

COBAS, plasma lactate measured with the laboratory method; PFO PL, plasma in fluoride oxalate, Accutrend in PL mode; PFO BL, plasma in fluoride oxalate, Accutrend in BL mode; WBFO PL, whole blood in fluoride oxalate, Accutrend in PL mode; WBFO BL, whole blood in fluoride oxalate, Accutrend in BL mode; WBHEP PL, whole blood in heparin, Accutrend in PL mode; WBHEP BL, whole blood in heparin, Accutrend in BL mode.

**FIGURE 2:**
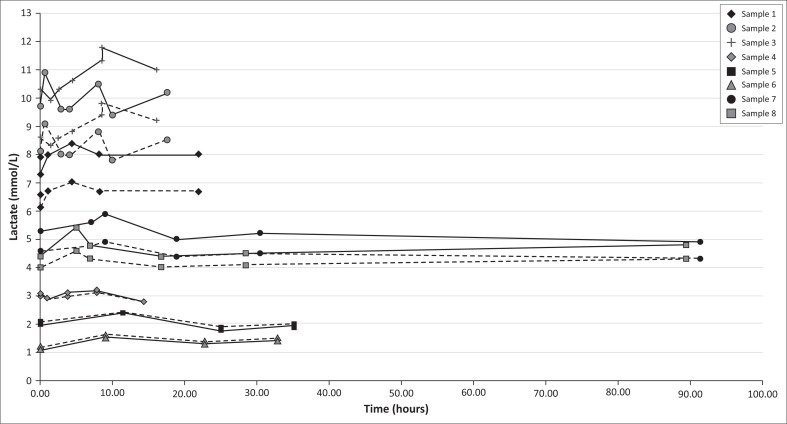
Lactate stability in sodium fluoride/potassium oxalate over time. The graph illustrates the measured concentration of lactate in 8 rhino blood samples (each sample designated by a shape marker) with sodium fluoride/potassium oxalate over time using the Accutrend meter set in either blood (BL, dashed line) or plasma (PL, solid line) mode.

The stability of lactate in fluoride/oxalate over time is shown in Figure 2. Six rhinos were included in the study (2 from reserve 3, 3 from reserve 2 and one from reserve 1). The two rhinos in reserve 3 were each sampled twice (3 months apart), so 8 samples were available in total. Lactate was measured on the Accutrend for each sample between four and seven times over a minimum of 14.3 to a maximum of 91.3 h. Lactate remained stable with a mean change (mean, 95% CI) from baseline to last measurement of 0.15 (−0.18, 0.48) mmol/L and 0.14 (−0.13, 0.4) mmol/L with the Accutrend in PL mode and BL mode, respectively (Table 3).

**TABLE 3:** Stability of rhino blood lactate in sodium fluoride/potassium oxalate over time.

Accutrend mode	*n*	Maximum change in blood lactate from baseline over time		Change in blood lactate from baseline at time 0, to last measurement	
		Max (mmol/L)	Mean, 95% CI (mmol/L)	Max (mmol/L)	Mean, 95% CI (mmol/L)
Accutrend in BL mode	8	1.2	0.39 (−0.08, 0.86)	0.6	0.14 (−0.13, 0.4)
Accutrend in PL Mode	8	1.5	0.53 (−0.08, 1.13)	0.7	0.15 (−0.18, 0.48)

Showing the absolute change in lactate from initial reading (baseline) to last reading, and the maximum change over time from baseline.

## Ethical considerations

This research (project number V059-12) was approved by the University of Pretoria Animal Ethics Committee. The ethical standards required in the University of Pretoria’s Code of ethics for researchers and the Policy guidelines for responsible research have been upheld throughout the research process.

Oral consent was given by the owners or caregivers of the rhinos for their inclusion in this study. Only rhinos being immobilised for management purposes were used in the study; so no animals were anesthetised solely for the purposes of research.

## Discussion

Bland–Altman analysis indicated good agreement between the Accutrend and the Cobas bench-top analyser when WBFO and PFO were used in both the PL and the BL mode. Moderate agreement was seen when WBHEP was used in both the BL and PL modes.

The highest level of agreement with a small bias, narrowest LOA and *Pc* closest to one was seen when WBFO was used on the Accutrend in the PL mode. When PFO was used on the Accutrend in PL mode, agreement with the Cobas bench-top analyser was also good with small bias, narrow LOA and *Pc* value close to one. For all sample types in this study (blood and plasma), the PL setting had better agreement with the Cobas bench-top analyser than the BL setting. A study evaluating the Accutrend in dogs also found that when whole blood was used the plasma setting provided better agreement with the reference method than the blood setting (Karagiannis et al. 2013). An equine study assessed the Accusport (which uses the same technology as the Accutrend) and found the highest level of agreement to a laboratory method when plasma was used in the plasma mode (Evans & Golland 1996). It has been suggested that the algorithm used by the Accusport and Accutrend analysers to convert measured plasma lactate values to blood values is accurate only for humans and may not be suitable for other animals because of differences in lactate distribution between blood cells and plasma (Evans & Golland 1996; Tennent-Brown et al. 2007). The results of the current study suggest that when measuring lactate in rhino blood or plasma on the Accutrend, the plasma mode may yield more accurate results.

The poorest agreement with the Cobas bench analyser was seen when WBHEP was used on the Accutrend, with wide LOA and *Pc* < 0.90 for both PL and BL modes. The Accutrend manufacturer’s instructions recommend the use of heparinised whole blood or fresh whole blood; and therefore anticoagulant choice is unlikely to affect results here. The reason for the relatively poor agreement seen with WBHEP in the current study may be related to sampling methods. At the time of sampling, blood was placed into two of each of plain, EDTA, heparin and fluoride/oxalate tubes. Extra blood was stored for future studies. In the time taken to fill all the tubes it is possible that the blood lactate concentration in the rhino may have changed. Studies indicate that lactate levels change over time during chemical immobilisation in rhinos, often decreasing in the initial period post immobilisation (Buss et al. 2015; Miller et al. 2013; Morkel et al. 2010) presumably as the metabolic acidosis caused by pre-capture exertion begins to normalise and because of the effects of butorphanol administration. The lactate concentration in the first tube filled may therefore be different from that in the last tube. As the order of tubes filled was random and not recorded, we were unable to test this hypothesis. As PFO was the sample type used for analysing lactate on the Cobas bench-top analyser, it may be that agreement with heparin samples are poor because of differences in lactate concentration between the heparin and fluoride/oxalate samples and not because of inaccuracy of the Accutrend. Lactate analysis on the Accutrend (in both BL and PL modes) using PFO was performed on the same day as the laboratory method analysis in a laboratory under controlled conditions, whereas analysis of WBHEP on the Accutrend was performed on several different days, under variable field conditions. It is possible that the effects of climate and inter-assay variation may have had a negative effect on agreement between methods. However, the WBFO analysis on the Accutrend was also performed in field conditions over several days, and when WBFO was used in the PL mode, agreement with the Cobas bench-top analyser was greater than for all other sample types. The sample size for the WBFO group was smaller and with a narrower range of lactate values than the other groups. With a larger sample size and a greater range of lactate values the differences between heparin and fluoride/oxalate samples may have been less marked. Owing to the instability of blood lactate in heparin and the impracticalities of separating plasma in the field, only heparinised whole blood was measured on the Accutrend, and it was not possible to assess agreement between the Accutrend and the Cobas bench-top analyser using heparinised plasma in this study.

Studies in horses have demonstrated improved accuracy of the Accusport and Accutrend when measuring lactate levels less than 10 mmol/L (Evans & Golland 1996; Schulman, Nurton & Guthrie 2012), or less than 5 mmol/L (Tennent-Brown et al. 2007) respectively. In the current study, tighter LOA were observed, with reasonable preservation of bias, when lactate was less than 5 mmol/L for all groups examined. Additionally, Passing and Bablok regression results indicated a lack of proportional and systematic differences for PFO PL, PFO BL, and WBHEP PL with lactate < 5 mmol/L. However, when concordance analysis was performed there was poorer agreement for all groups when split according to low or higher lactate concentration than with results combined. The majority of blood lactate values measured in this study were < 10 mmol/L and larger sample size and a greater range of lactate values would be needed to properly assess the performance of the Accutrend at higher lactate values. Although the results of this study indicate that the Accutrend may have better accuracy when measuring lower lactate levels, the Accutrend results will likely be sufficient to follow trends when measuring lactate in rhinos in the field. When assessing a new method of measuring a clinical variable, an important point to consider is if the new method is interchangeable with the old method over the clinically relevant range of measurements (Altman 2009). The clinically relevant range in the rhino has yet to be determined; however, the Accutrend had a good level of agreement with the Cobas bench-top analyser over the range of lactate values tested here (0.8 mmol/L – 16.5 mmol/L in PL mode).

For this device to be of use to veterinarians treating rhinos, it must be suitable for use in field conditions. The instruction manual for the Accutrend states that for lactate testing the instrument must be operated between 15 °C and 35 °C, on a level surface. On one occasion during the study, ambient temperatures exceeded 35 °C. The device indicated a temperature error. This was an advantage as it meant that it was not possible to record an erroneous result when the machine was outside of its temperature range. The field conditions in this study were variable and the device was at times operated on the back of a vehicle moving over rough terrain. Lactate in whole blood was measured in the field in this study (plasma was tested in the laboratory), and when the device was in PL mode there was good agreement with the Cobas bench-top analyser. Overall the device performed well in the field in this study and is therefore likely to be suitable for field measurement of lactate in the white rhino providing the manufacturer’s limitations are observed.

Continuation of cellular glycolysis after venepuncture results in a rise in blood lactate levels over time if blood is kept in tubes containing heparin or EDTA (Astles, Williams & Sedor 1994). The addition of sodium fluoride/potassium oxalate (an antiglycolytic agent) maintains stable lactate levels in human blood at room temperature for 8 h (Astles et al. 1994) or in equine blood refrigerated for up to 6 h (Tennent-Brown et al. 2010; Williamson et al. 1996). The rhino blood samples in this study were stored for 14 h to 91 h under highly variable conditions. Field temperatures ranged from 22 °C to > 35 °C, and although blood tubes were stored in cooler bags with ice, it was often not possible to replenish ice regularly meaning blood storage temperatures for the first 4–10 h in the field would have been variable. The maximum change in blood lactate over time in this study was 1.5 mmol/L for the PL setting with a mean (95% CI) change of 0.53 (−0.08, 1.13) mmol/L (Table 3). The mean change is small and is unlikely to be clinically significant. A maximum change of 1.5 mmol/L could be clinically significant at lower lactate levels; however, this maximum difference of greatest magnitude was seen in a sample with a high initial lactate level, and although not fully assessed here it can be seen from the graph (Figure 2) that when lactate levels were low the changes over time were minimal (< 0.6 mmol/L for all lactate values < 4 mmol/L) suggesting that this would not interfere with diagnosing a clinically important hyperlactaemia at lower lactate levels. The overall mean (95% CI) change for all samples from baseline to final measurement was 0.15 (−0.178, 0.478) mmol/L. These results are similar to those in a study that reported a mean increase of 0.15 (0–0.3) mmol/L in controlled laboratory conditions with human samples kept in fluoride/oxalate for 24 h at room temperature (Astles et al. 1994).

The apparent stability of rhino blood lactate in fluoride/oxalate tubes over a long period and in variable temperature conditions has important practical implications for clinicians wishing to measure blood lactate in the field where a portable handheld lactate meter is not available.

Normal resting values for blood lactate in white rhinos (and other rhino species) without the effects of pre-capture exertion or respiratory depressant immobilising drugs are currently not available in the literature. There were variable levels of pre-capture exertion in this study, and the effects of butorphanol and time from darting which are factors that have been shown to effect lactate levels in rhinos (Buss et al. 2015; Miller et al. 2013) have not been examined here. In addition, a range of lactate values was desirable in this study to assess the accuracy of the Accutrend at low and high lactate values. The utility of lactate testing in the field and the ability of blood lactate levels to predict outcome in critically injured rhinos still need to be evaluated. Lactate levels greater than 2 mmol/L would be considered elevated in humans (Levy 2006), 1.5 mmol/L is the upper limit of normal in horses (Tennent-Brown 2011), whereas dogs have normal values of 0.3 mmol/L – 2.5 mmol/L (Sharkey & Wellman 2013). These are fairly narrow ranges of normal lactate, which seem to be conserved across mammalian species, and rhinos may well have a similar range. As lactate in free-ranging rhinos is only measured when rhinos are immobilised it will be difficult to differentiate elevated lactate levels caused by disease, from transient elevations caused by exertion or immobilising drugs. Studies in humans (Husain et al. 2003; McNelis et al. 2001; Nguyen et al. 2004) and horses (Johnston et al. 2007; Tennent-Brown et al. 2010; Wotman et al. 2009) have found that serial lactate levels may be more useful in predicting outcome and monitoring response to treatment than single measurements. Critically injured rhinos are now being immobilised repeatedly for treatment and re-assessment of injuries. Some are being boma restricted or confined to smaller areas to make management easier. In these circumstances, serial lactate measurements may be possible. A handheld meter such as the Accutrend evaluated in this study could be a suitable field tool for this sort of investigation. As results are available in a short time period and test strips are inexpensive, it may also be a useful tool for physiological monitoring during immobilisation; in field or captive situations in conjunction with other observed clinical parameters such as heart or respiratory rate and monitoring tools such as pulse oximetry.

## Conclusion

The Accutrend system provided results in close agreement to those measured on a laboratory based analyser when whole blood or plasma in fluoride/oxalate was used with the device set in plasma mode. It is light, portable, easy to operate in field conditions and is suitable for field use in white rhinos. Rhino blood lactate is stable in sodium fluoride/potassium oxalate for as long as 3 days, this has implications for veterinarians working in remote locations who wish to measure lactate.
